# Evaluation of the Implementation and Effectiveness of a Mobile Health Intervention to Improve Outcomes for People With HIV in the Washington, DC Cohort: Study Protocol for a Cluster Randomized Controlled Trial

**DOI:** 10.2196/37748

**Published:** 2022-04-22

**Authors:** Jacqueline Hodges, Sylvia Caldwell, Wendy Cohn, Tabor Flickinger, Ava Lena Waldman, Rebecca Dillingham, Amanda Castel, Karen Ingersoll

**Affiliations:** 1 Division of Infectious Diseases and International Health University of Virginia Charlottesville, VA United States; 2 Public Health Sciences University of Virginia Charlottesville, VA United States; 3 Department of Medicine University of Virginia Charlottesville, VA United States; 4 Milken Institute School of Public Health George Washington University Washington, DC United States; 5 Department of Psychiatry and Neurobehavioral Sciences University of Virginia Charlottesville, VA United States

**Keywords:** human immunodeficiency virus, HIV, mobile health, mHealth, implementation science, cluster randomized controlled trial, smartphone

## Abstract

**Background:**

Gaps remain in achieving retention in care and durable HIV viral load suppression for people with HIV in Washington, DC (hereafter DC). Although people with HIV seeking care in DC have access to a range of supportive services, innovative strategies are needed to enhance patient engagement in this setting. Mobile health (mHealth) interventions have shown promise in reaching previously underengaged groups and improving HIV-related outcomes in various settings.

**Objective:**

This study will evaluate the implementation and effectiveness of a clinic-deployed, multifeature mHealth intervention called PositiveLinks (PL) among people with HIV enrolled in the DC Cohort, a longitudinal cohort of people with HIV receiving care in DC. A cluster randomized controlled trial will be conducted using a hybrid effectiveness-implementation design and will compare HIV-related outcomes between clinics randomized to PL versus usual care.

**Methods:**

The study aims are threefold: (1) We will perform a formative evaluation of PL in the context of DC Cohort clinics to test the feasibility, acceptability, and usability of PL and tailor the platform for use in this context. (2) We will conduct a cluster randomized controlled trial with 12 DC Cohort clinics randomized to PL or usual care (n=6 [50%] per arm) and measure the effectiveness of PL by the primary outcomes of patient visit constancy, retention in care, and HIV viral load suppression. We aim to enroll a total of 482 participants from DC Cohort clinic sites, specifically including people with HIV who show evidence of inconsistent retention in care or lack of viral suppression. (3) We will use the Consolidated Framework for Implementation Research (CFIR) and the Reach Effectiveness Adoption Implementation Maintenance (RE-AIM) framework to measure implementation success and identify site, patient, provider, and system factors associated with successful implementation. Evaluation activities will occur pre-, mid-, and postimplementation.

**Results:**

Formative data collection was completed between April 2021 and January 2022. Preliminary mHealth platform modifications have been performed, and the first round of user testing has been completed. A preimplementation evaluation was performed to identify relevant implementation outcomes and design a suite of instruments to guide data collection for evaluation of PL implementation throughout the trial period. Instruments include those already developed to support DC Cohort Study activities and PL implementation in other cohorts, which required modification for use in the study, as well as novel instruments designed to complete data collection, as guided by the CFIR and RE-AIM frameworks.

**Conclusions:**

Formative and preimplementation evaluations will be completed in spring 2022 when the trial is planned to launch. Specifically, comprehensive formative data analysis will be completed following data collection, coding, preliminary review, and synthesis. Corresponding platform modifications are ready for beta testing within the DC Cohort. Finalization of the platform for use in the trial will follow beta testing.

**Trial Registration:**

ClinicalTrials.gov NCT04998019; https://clinicaltrials.gov/ct2/show/NCT04998019

**International Registered Report Identifier (IRRID):**

PRR1-10.2196/37748

## Introduction

### Background

Despite progress made toward addressing the HIV epidemic within the United States, concerted efforts are needed to address substantial gaps in the care continuum for people with HIV [1]. Retention in HIV care and achievement and maintenance of viral suppression are critical steps of the continuum [2]; however, currently less than half of all people with HIV in the United States are considered retained in care, and even fewer have achieved viral suppression [3,4]. Missed outpatient visits are an important early marker of failure to achieve suppression and are associated with increased mortality [5-7].

National gaps persisting within the HIV care continuum are similar for people with HIV in Washington, DC (hereafter DC), which is a priority jurisdiction of the Ending the Epidemic Initiative [8]. Specific subpopulations of people with HIV have previously demonstrated higher discontinuity of care, including those who are male, Black, younger, uninsured, and with injection drug use as a risk factor for exposure to HIV [9,10]. People with HIV in DC face various barriers to retention in care, including limited transportation, lack of comprehensive medical case management and adherence-related services, absence of a medical home, and gaps in health literacy [11,12].

### The DC Cohort

The DC Cohort is the largest citywide prospective cohort of people with HIV in the United States, with 11,700 participants having consented at 15 partnering clinics across DC [13]. The DC Cohort Study is a National Institutes of Health (NIH)-funded study conducted in partnership between the DC Center for AIDS Research (CFAR), DC Department of Health (DOH), and the NIH/National Institute of Allergy and Infectious Diseases (NIAID) as part of the DC Partnership for AIDS Progress [12]. Among the 15 sites, 14 (93%) agreed to participate in the study at the time of funding.

DC Cohort clinics range in size, characteristics, patients, and populations served and include federal, academic, and community-based clinics, as well as pediatric and adult clinics. Through a network of research assistants (RAs) located at these partner clinics, approximately 450 people with HIV enroll in the DC Cohort annually. DC Cohort participants represent about 75% of people with HIV cared for at these clinics and are demographically similar to the broader HIV population in DC; in 1 interim assessment, 4258 (82%) of 5193 participants were black, 3531 (68%) male, and 1973 (38%) men who have sex with men (MSM) [14].

### Mobile Health and HIV

Smartphone accessibility is high overall in the United States [15]. Mobile health (mHealth) interventions developed to encourage self-management and social support and enhance the mental health of people with HIV have been associated with improvement in antiretroviral therapy (ART) adherence and retention in care for at-risk groups [16-25]. A range of retention-in-care services are offered across DC Cohort sites [26,27]; however, no mHealth interventions have been systematically deployed or studied within these sites.

**Figure 1:**
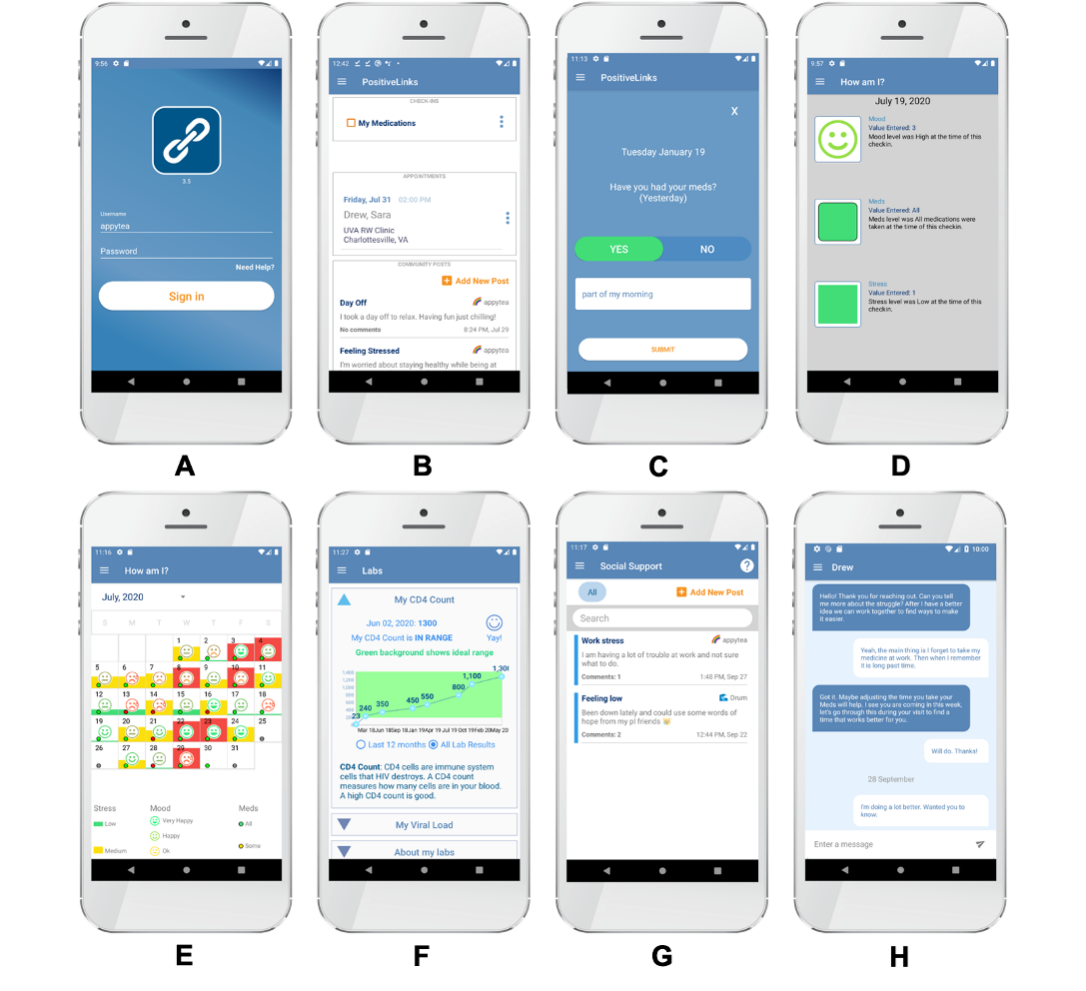
Screenshots of the home page and several features of PL. Features shown include a discreet home screen (A) and a dashboard (B), daily queries for medication administration (C), and stress and mood (D), along with discreet push notifications. The “How am I?” screen (E) provides graphical feedback based on user answers to daily queries for self-monitoring. Additional features include a lab results page (F), an anonymous community message board (G), and secure in-app messaging between patients and providers/staff (H). Users can also upload documents that support Ryan White eligibility. PL: PositiveLinks.

*PositiveLinks* (PL) is a clinic-associated mHealth platform available to providers (in outpatient settings, including clinical and nonclinical) and patients through a smartphone app. Clinic providers also access a web portal to manage patient cohorts, an online implementation guide, and an online learning management system for training and certification. PL features were designed using psychological theories of behavior change (information-motivation-behavioral skills model [28] and social action theory [29]) and principles of motivational interviewing that encourage self-directed behavior change [30], and are informed by user-based design [31-38]. The PL platform delivers appointment reminders; daily queries (“check-ins”) of mood, stress, and medication adherence with self-monitoring feedback; display of recent cluster of differentiation (CD)4 and viral load lab results; access to PL support staff for assistance/troubleshooting; secure communication with providers and clinic staff using in-app messaging; tailored educational resources; and the ability to interact with other users on a secure, anonymous community message board (Figure 1). The PL platform has also been adapted and translated for use in Spanish-speaking populations [39].

A prospective study of poorly retained people with HIV in Virginia found that PL usage increased retention in care and improved the cohort rate of viral suppression by over 30% at 12 months [40]. Additionally, PL was included as an evidence-based strategy to improve engagement in care for people with HIV in a 2020 update to guidelines by the HIV Medicine Association of the Infectious Diseases Society of America [41]. Although PL is a promising tool for people with HIV, efficacy has not been evaluated with a rigorous randomized trial or in urban populations. We describe a protocol for a cluster randomized controlled trial using a type II hybrid effectiveness-implementation design to test PL against usual care for people with HIV receiving outpatient HIV care in the DC Cohort.

The aims of this study are threefold. We first aim to determine the feasibility, acceptability, and usability of PL within DC Cohort clinic sites in order to tailor the PL platform for use in this context. Our second aim is to determine the effectiveness of PL in relation to key clinical outcomes of viral suppression, visit constancy, and retention in care. Our third aim is to use validated implementation science frameworks to rigorously measure the success of implementation of the PL program within participating DC Cohort sites and identify site, patient, provider, and system-level factors critical for successful implementation.

## Methods

### Outcome Measures

Primary outcomes to be evaluated at 12 months following participation include viral suppression (HIV viral load <200 copies/mL), visit constancy (proportion of 4-month time intervals, with 1 visit with an HIV care provider completed in the 12-month period of study participation), and retention in care by the Health Resources and Services Administration (HRSA)-1 measure (keeping 2 HIV care appointments separated by 90 days within the 12-month time period of study participation) [42].

The primary hypotheses to be tested are that compared to participants at DC Cohort sites randomized to usual care, participants at sites randomized to receive the intervention (tailored PL platform with associated program activities) will demonstrate, on average:

- 15% better viral suppression at 12 months
- 25% greater rate of visit constancy at 12 months
- 25% greater rate of retention in care at 12 months

The study will also test the impact of PL versus usual care on secondary outcomes related to patient psychosocial characteristics, including markers of mental health, stigma, social support, and drug use. Finally, relevant implementation-centered outcome measures identified during the preimplementation evaluation (described in further detail later) will be evaluated during the mid- and postimplementation phases of the study.

### Study Aim 1: Formative Evaluation

To determine the feasibility, acceptability, and usability of PL within DC Cohort clinics, we performed a series of focus groups and in-depth interviews with stakeholders.

#### DC Regional Planning Commission, DC Cohort Executive Committee, and DC Cohort Site Providers

Focus groups were conducted with members of the DC Cohort Executive Committee (DC Cohort site principal investigators (PIs), NIH and DOH representatives; n=10) and the DC Regional Planning Commission on Health and HIV (COHAH; n=50). Both focus groups met online during COVID-19 surges in DC. Members were included in focus groups based on availability to participate; all members were invited to participate and provided informed consent. In-depth interviews were also conducted with 2 providers from 14 DC Cohort sites (eg, clinicians, nurses, case managers, social workers, and support staff; n=28).

Provider focus groups were semistructured. An interview guide designed by the study team was used to collect perspectives on barriers to and facilitators of retention in care and viral suppression, input on app features based on experiences with the population of people with HIV they serve, and potential modifications felt to be most useful for enhancing retention in care. In-depth interviews were also semistructured and conducted using the same interview guides.

#### People With HIV Receiving Care in the DC Cohort

Focus groups were also conducted with a subset of people with HIV from 14 DC Cohort sites (5 focus groups, n=32 patients representing 8 sites). Eligible people with HIV were those who were aged 16 years or older, were receiving care at a DC Cohort clinical site, spoke English, could provide legal informed consent, and could participate virtually (due to the COVID-19 pandemic restrictions). Participants were identified, recruited, and consented from DC Cohort sites by site RAs. Participants were remunerated with a US $25 gift card. Individual think-aloud user testing has also been completed (approximately 14/482 [2.9%] expected to enroll). COVID-19 precautions were observed during all sessions. Participants were remunerated with US $50 gift cards and US $10 metro cards. Following the user testing phase, 1 DC Cohort site withdrew from the study and 2 DC Cohort clinics merged, leaving 12 active sites. The site that withdrew cited staffing issues during the COVID-19 era that would present a challenge to fully participate. After user testing, final beta testing to detect any bugs, glitches, or data loss issues will be conducted with an additional 14 participants who will use the DC Cohort PL platform with assistance from their clinic RA for 1 month. People with HIV who participate in the beta testing will be remunerated with a US $50 gift card and a US $10 metro card for each session.

#### Focus Group Testing Among People With HIV in the DC Cohort

Focus groups were conducted using semistructured interview guides designed to elicit patient knowledge and perspectives surrounding engagement in care and viral suppression, assess comfort with and use of technology and smartphone apps, and elicit perceptions about PL and its potential role in supporting engagement in care. Following demonstration of the PL platform features, interviewers elicited feedback on interest in the app and preferences for particular app features. Brief surveys were distributed at the end of the focus groups and included questions relating to self-reported adequacy of retention in care and care-seeking behaviors, unmet needs, comorbidities, perceptions of the patient-provider relationship, and levels of user experience/comfort with smartphones.

Themes elicited from focus group and in-depth interviews were synthesized by the study team and presented to the DC Cohort Executive Committee, and consensus was reached on app modifications. Requested modifications were then provided to the PL development team.

#### Think-Aloud User Testing Among People With HIV in the DC Cohort

The modified app was iteratively tested with DC Cohort participants using the think-aloud protocol [43,44]. In total, 14 users provided input during 1-hour task-focused individual sessions and completed surveys at the end of the session, demonstrating how they navigate app features, while voicing their opinions about their experience of PL.

#### Beta Testing Among People With HIV in the DC Cohort

Investigators and developers discussed modifications to the app based on formative work following a preliminary review. The development team made modifications agreed on by the team. Beta testing of the near-finalized app will be conducted with 14 people with HIV, and the participating DC Cohort site RAs will be assigned to oversee patient enrollment, training on PL use, and ongoing app troubleshooting for the study. Participant interviews will be performed to solicit input after the first week of PL use in order to identify any issues with logins, navigation, functionality, or technical issues. Interviews will be repeated after the 1-month testing period concludes. A postparticipation survey will be performed to elicit feedback on the participants’ usage of the app over the 1-month period using the System Usability Scale [45]. Paradata metrics collected automatically by the app will be reviewed for the period as well. The research and PL development team will review all output from beta testing and make final app modifications.

#### Formative Data Analysis

All focus groups and interviews were audio-recorded and transcribed verbatim. Analysis of text files will be completed in spring 2022 using qualitative analysis software (Dedoose) with an a priori open coding process to identify themes and categories. Coding will be performed by at least 2 independent RAs to achieve consensus. Descriptive statistics will be used to analyze participant survey data for focus groups, in-depth interviews, and user and beta testing.

### Study Aim 2: Cluster Randomized Controlled Trial

**Figure 2:**
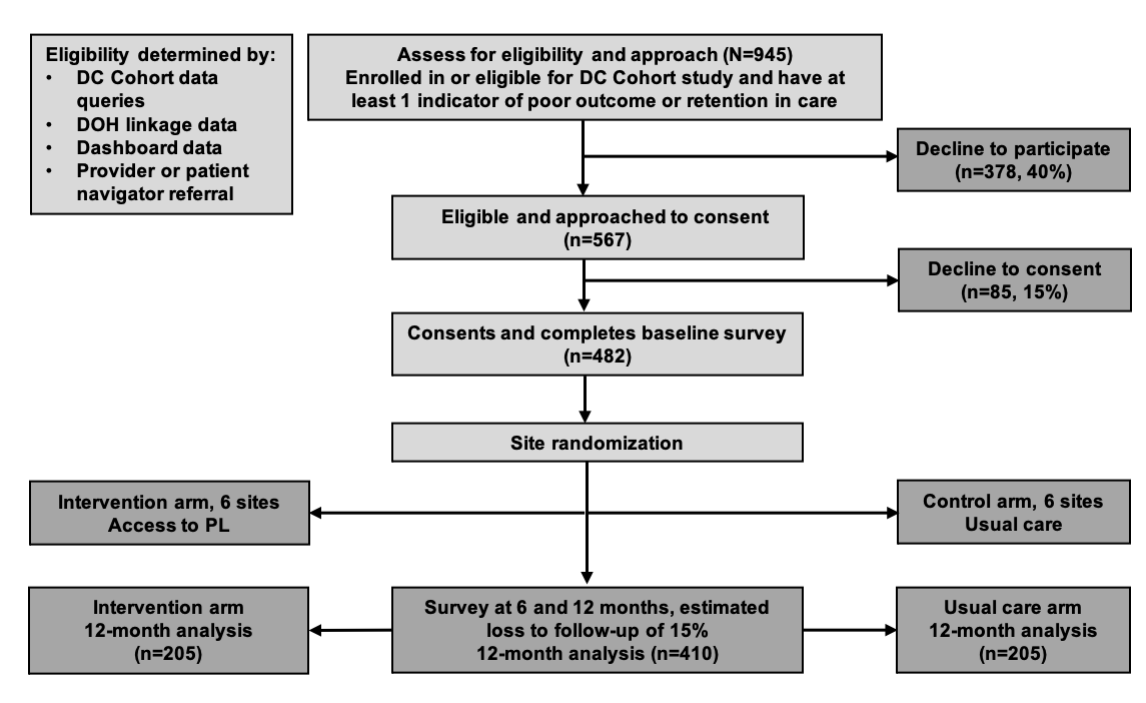
Cluster randomized controlled trial recruitment and participant flow diagram. DC: Washington, DC; DOH: Department of Health; PL: PositiveLinks.

This study is a cluster randomized controlled trial whereby 12 clinics will be randomized to PL (n=6, 50%) or usual care (n=6, 50%); see Figure 2. Participants from clinics randomized to PL will receive access to the smartphone app following training on use provided by the site RA. Clinic providers (ie, clinicians, nonclinical care providers, support staff) will have access to the provider online learning management system for training on PL use, the PL provider online portal and smartphone app, and remote assistance provided by the PL program team. On-site administration will be supervised by site RAs. Patients at clinics randomized to PL will be able to use the app for at least 12 months up to the date of trial completion. Participants from clinics randomized to usual care will receive usual clinic retention and medication adherence support services for 12 months. Trial activities will complete in 2025.

#### Recruitment

Participants in the cluster randomized controlled trial will be recruited from 12 clinics participating in the DC Cohort Study. Informed consent will be obtained for all DC Cohort participants under a protocol approved by the George Washington University Institutional Review Board (Protocol NCR202829; ClinicalTrials.gov NCT04998019). Inclusion criteria are people with HIV who (1) are enrolled in the DC Cohort study; (2) are aged 16 years or more; (3) if a minor, are in charge of their own HIV care (with waiver of parental consent); (4) speak and read English or Spanish at the fourth-grade level or above; (5) can provide informed consent; (6) plan to reside in the DC metro area for 12 months following enrollment; and (7) have at least 1 of the following putative indicators of poor retention (in order of priority): (1) detectable viral load, (2) not retained in care, (3) returning to care after a gap of ≥6 months, (4) no visit constancy in the 12 months prior to enrollment, (5) newly diagnosed or initiating HIV care, (6) recently transferred from a different HIV care site, or (7) evidence of simultaneous HIV care receipt at a DC Cohort site and a non-DC Cohort site based on DC DOH surveillance data. Exclusion criteria include people with HIV who are (1) aged below 16 years or if 16-17 years old have a parent in charge of HIV care and (2) unable to provide informed consent. To minimize cross-site contamination, people with HIV receiving care at 2 DC Cohort sites will be excluded. Eligible patients will be identified by the study team with monthly review of the DC Cohort database as well as input from site providers, patient navigators, and RAs. Patients who do not own a smartphone on enrollment will be provided a study smartphone to use for the duration of the study.

#### Randomization

In total, 6 clinics will be randomized to the intervention arm, and 6 clinics will be randomized to the usual care arm. Randomization will maximize the balance between arms in terms of clinics’ predominant patient population characteristics (adolescent vs adult), panel size (eg, the 2 largest clinics will be randomized, 1 to each arm), and availability of Ryan White Clinical services, with all other clinics randomized to each arm by the study statistician. Given the nature of the intervention, neither researchers nor participants will be blinded to the outcome of randomization of clinic sites.

#### Sample Size Determination

Sample size calculation and power analysis are based on data from the PL 12-month prospective outcome study [40] showing a 30% increase in viral suppression at 12 months and on DC Cohort data showing that 55% of participants achieved viral suppression within a 12-month period [27]. We would need a mean cluster size of 64 or 768 participants to detect a 15% difference. We would need to enroll 432 participants to detect a more conservative 17.5% increase in viral suppression at 12 months, for 80% power to detect a true difference between PL and usual care as 36 per condition, assuming an intraclass correlation coefficient of .02 and a coefficient of variation of cluster sizes of 0.5. To achieve a sample size with sufficient power, we aim to enroll a total of 482 participants.

We estimate 60% of the people with HIV approached will be interested in the study based on the mean study consent rates for prior DC Cohort studies [46,47]. We plan to approach 945 people with HIV and enroll 482 (51%) participants overall (approximately n=40, on average, per cluster). Based on prior experience in recruiting people with HIV for studies in DC, recruiting 482 people with HIV over 20 months at the 12 clinics (at least 2 people per clinic per month) will be achievable, given the percentage of DC Cohort participants (7839/11,700, 67%) meeting the inclusion criteria. Based on previous experiences with PL and mHealth interventions [16,40], we anticipate approximately 15% participants lost to follow-up, resulting in 410 (85.1%) of 482 participants for analysis at the study endpoint.

#### Trial Data Collection

At study enrollment, baseline surveys will be completed by trial participants to evaluate specific sociodemographic measures relevant to retention in care [9,10], including age, sex, race, injection drug use, social determinants of health (eg, food insecurity, financial and housing instability), and changes in contact information in the past year. At study enrollment, 6 and 12 months, patients will be surveyed on risk factors for poor retention or lack of viral suppression, including medication nonadherence, self-efficacy, depression, and stressful life events [48] and psychosocial measures relevant to outcomes in PL studies, including experiences with stigma [49], social support, mental health, perceived stress, quality of life, self-efficacy related to substance use, and patient-provider communication. The site RA will administer baseline surveys using REDCap software during the enrollment visit, while 6- and 12-month surveys will be administered at a clinic visit or over the telephone. All data will be entered into REDCap.

#### Efficacy Outcomes

All DC Cohort sites have RAs designated to collect and export patient laboratory values (eg, CD4 count, HIV viral load), sociodemographics, comorbid diagnoses, and encounter data into a central database (DC Cohort Study Database). Data exported for our study will be restricted to a 45-day window surrounding each participant’s baseline, 6-month, and 12-month dates.

#### Characterizing Usual Care in DC Cohort Clinics

A standardized site assessment form was distributed as part of DC Cohort Study activities in 2016 to characterize the range of services comprising usual care delivered by DC Cohort clinic sites [27]. Services queried include clinic staffing (size, provider training, work experience), on-site clinical services, and activities to support patient ART adherence or linkage and retention in HIV care. Based on this assessment, the usual care condition across sites ranges from no ancillary support to comprehensive services (case management, adherence support, patient navigation, mental health, substance use, dental services, and food banks). This site assessment form will be redistributed electronically via REDCap to all sites (n=12, 100%) prior to initiation of the trial.

#### Trial Statistical Analysis

To compare the primary outcomes of viral suppression, visit constancy, and retention in care at 12 months between clusters, we will perform logistic regression using a mixed effects model (MEM), accounting for heterogeneity, including unequal cluster size between sites and correlations between participants from the same clinic site and between repeated measurements on the same participant group [50-52]. The comparisons between the conditions will be adjusted for both individual-level (gender, race, age, mode of HIV transmission) and cluster-level (differences in availability of specific adherence, retention and counseling services provided by sites) characteristics.

The MEM will also compare each secondary outcome of interest (psychosocial characteristics at 6- and 12-month follow-up assessments) between clusters within the PL and usual care arms. Linear regression and logistic regression based on MEMs will be used to analyze continuous outcomes and binary outcomes, respectively, adjusted for the same individual- and cluster-level characteristics as with primary outcome analyses.

#### Adverse Events

Site RAs will routinely monitor and moderate activity on the PL platform by the respective site’s patient panel, including any inflammatory content or disclosure of identifying information posted on the community message board. Should such activity be identified, procedures to notify the site PIs will be instituted to determine whether additional actions are necessary to prevent further dissemination of inappropriate content.

#### Ethics Approval

Informed consent will be obtained for all trial providers and participants using an approved protocol, with ethical approval provided by the George Washington University Institutional Review Board (Protocol NCR202829; ClinicalTrials.gov NCT04998019).

### Study Aim 3: Implementation Evaluation

Implementation of PL will be evaluated in parallel with efficacy of the intervention. Validated implementation science frameworks will be used pre-, mid- and post-PL implementation to determine factors that influence the relative success of the implementation strategy used to recruit, train, and retain patients and providers in the PL intervention. Specifically, we used the Reach Effectiveness Adoption Implementation Maintenance (RE-AIM) framework [53] to identify relevant implementation-centered outcomes during the preimplementation evaluation and will apply the framework mid- and postimplementation to measure and compare implementation success at each step of the implementation strategy executed across DC Cohort sites. The Consolidated Framework for Intervention Research (CFIR) [54] also guided preimplementation activities, as described later, and will be applied mid- and postimplementation toward identification of relevant barriers and facilitators of implementation success within DC Cohort sites at the patient, provider, clinic, and broader organizational levels.

#### Preimplementation Evaluation

**Figure 3:**
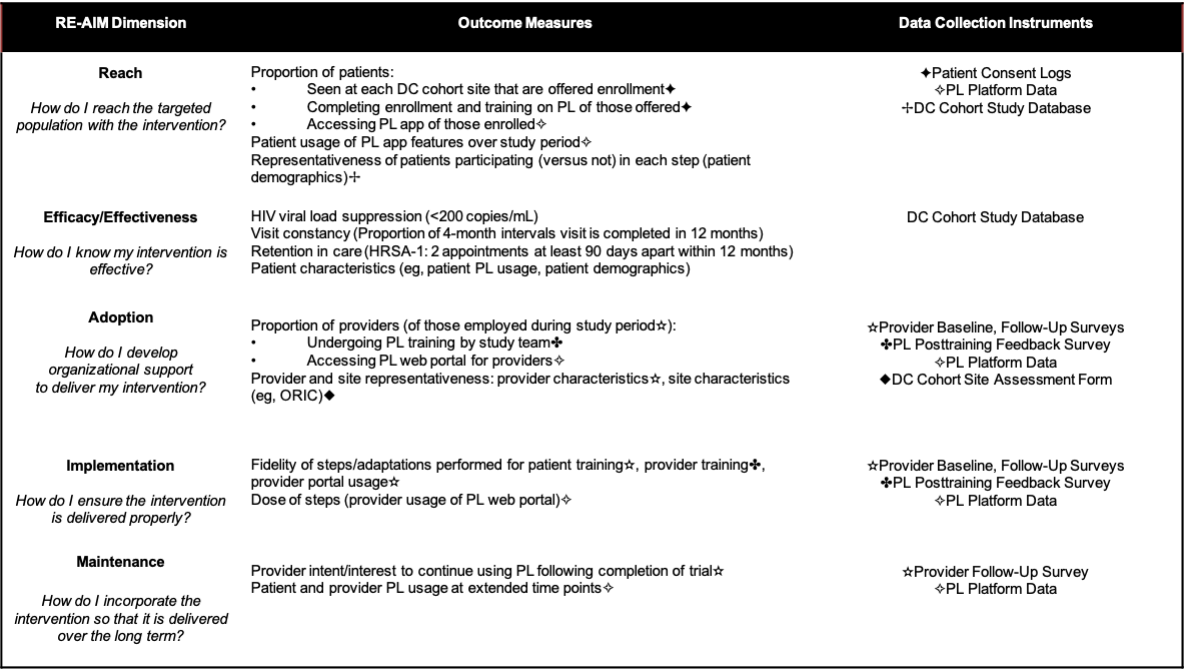
RE-AIM framework dimensions. Dimensions of interest for PL implementation in DC Cohort clinics are listed along with corresponding outcome data requiring collection, as well as the instruments that will be utilized. For each framework dimension, outcome measures evaluated using multiple different instruments are denoted with a corresponding symbol matched to the instrument used. DC: Washington, DC; HRSA: Health Resources and Services Administration; ORIC: Organizational Readiness for Implementing Change; PL: PositiveLinks; RE-AIM: Reach Effectiveness Adoption Implementation Maintenance.

During the preimplementation period, the DC Cohort Executive Committee, DC Cohort site leadership, and provider concerns related to the process of PL implementation were evaluated in conjunction with formative phase activities (focus groups, in-depth interviews). The output of both rounds of user testing conducted with people with HIV during the formative phase is currently being analyzed for any implementation-related concerns. During the preimplementation evaluation, we also used the RE-AIM framework to identify data points required to evaluate PL implementation based on predefined outcomes of interest across sites (Figure 3). A suite of instruments was then developed to support data collection to adequately capture all identified outcomes of interest and corresponding data points.

#### Mid- and Postimplementation Evaluations

During the mid- and postimplementation evaluations, we will conduct in-depth interviews with a subset of site providers and RAs (n=24). Postimplementation, an additional focus group will be repeated with the DC Cohort Executive Committee. We will also conduct 4 focus groups postimplementation with a subset of PL trial participants (32/482 [6.6%] expected to enroll). People with HIV will be sampled to ensure demographic diversity and include a range of users (eg, frequent vs infrequent users) who will be remunerated with a US $25 gift card and a US $10 metro card. For each interview/focus group conducted mid- and postimplementation, a semistructured interview guide will be used, developed based on our prior application of the CFIR to evaluate PL implementation [55]. Postimplementation interview guides will be updated in an iterative fashion based on analysis of midimplementation feedback.

#### Implementation Evaluation Data Collection

A suite of data collection instruments relevant to our implementation evaluation and planned for deployment to stakeholders mid- and postimplementation were developed, as described during the preimplementation evaluation (Table 1). Informed consent to participate in the implementation evaluation phase of the study will be obtained during the trial consent process for patients. Providers will be offered open enrollment in PL across sites randomized to the intervention, and informed consent will be obtained to participate in implementation evaluation activities.

**Table 1:** Data collection instruments identified during the preimplementation phase to support collection of relevant data points for the implementation evaluation phase of cluster randomized controlled trial.

Instrument	Description of instrument	Frequency and timing of data collection and export
DC^a^ Cohort Study Database	Patient laboratory values, encounter data, and sociodemographic data are routinely collected for all people with HIV in the DC Cohort.	Data are collected continuously by site RAs^b^ and uploaded/exported to the database monthly for all people with HIV in the DC Cohort, including trial participants.
Patient consent logs	Patient responses are tracked to site RAs, who consent and enroll participants in DC Cohort studies. These logs will be modified for use in the trial to track patient enrollment and completion of various steps of PL^c^ program implementation.	Log responses are uploaded and updated within the DC Cohort Study Database by RAs monthly.
DC Cohort site assessment forms	Site assessment forms query various site-level characteristics and will be modified to include items related to PL implementation (eg, site-level use of telemedicine, other mHealth^d^ tools). Forms will be deployed electronically and completed by site PIs^e^ at the start of the study period.	Interim distribution of the site assessment forms to site leadership (to be completed with site staff/provider assistance) will be performed throughout the study period at a yearly interval.
Provider baseline and follow-up surveys	Surveys will assess provider characteristics of interest, including specialty, time employed at the site, specific training, and baseline technology use. Follow-up survey items include perspectives on provider roles within the program, program adaptations, and individual usage of PL.	Baseline surveys will be distributed to site providers at the start of the study period and then every 6 months to providers newly employed during the study period and consenting to participation. Follow-up surveys will be redistributed to providers at 6-month intervals throughout the study period.
PL posttraining feedback survey	Providers completing the training step of PL implementation will be tracked by completion of a posttraining survey. Feedback elicited will include perceptions of the online learning management system (eg, modules). The survey was modified to include items from the ORIC^f^ measure.	An electronic feedback survey immediately follows completion of learning modules via the online learning management system. Survey responses will be exported for mid- and postimplementation evaluations.
PL paradata	Platform paradata metrics include user logins, screens viewed, features used, and screen time. In-app content includes patient responses to daily queries, messages posted on the community message board, and secure messages exchanged between patients and clinic providers.	Paradata metrics are collected and stored in the platform automatically and continuously. In-app content will be exported for analysis for the postimplementation evaluation.

^a^DC: Washington, DC.

^b^RA: research assistant.

^c^PL: PositiveLinks.

^d^mHealth: mobile health.

^e^PI: principal investigator.

^f^ORIC: Organizational Readiness for Implementing Change.

Existing data instruments were identified, including those developed to support the broader DC Cohort Study (DC Cohort Study Database, patient consent logs, site assessment forms), as well as those developed and used for evaluations across different clinic sites implementing PL in various contexts (PL posttraining feedback survey, PL paradata). Modifications to these instruments were planned to further support collection of specific data points required to capture all RE-AIM outcome measures that were elucidated during the preimplementation phase. For example, the PL posttraining feedback survey follows providers’ completion of learning modules included in the online learning management system used to train them on how to use PL. For providers in this trial, we plan to modify the survey to include validated items from the Organizational Readiness for Implementing Change (ORIC) measure [56], which assesses providers’ perceptions of the collective psychological readiness of their DC Cohort sites to implement organizational changes necessary to incorporate the PL program within their clinic’s activities (a characteristic that can be examined at the level of an individual provider and in aggregate at the site level, which is important for the “adoption” dimension of RE-AIM).

Novel instruments were also designed to complete necessary data collection for corresponding RE-AIM dimensions, including both the provider baseline and provider follow-up surveys. Provider follow-up surveys, for example, probe for shifting roles and adaptations related to program implementation made by providers, different ways in which providers engage with available PL features, and provider perspectives on materials and processes supporting program implementation within the DC Cohort sites (all relevant to the “implementation” dimension of RE-AIM).

#### Analysis of Implementation Outcomes

All interviews and focus groups conducted for implementation evaluations will be audiotaped, transcribed, and analyzed using Dedoose, as described for formative data analysis. We will use a deductive approach built around CFIR constructs for analysis of stakeholder interviews, with specific constructs selected from our prior evaluation [55] as the a priori categories to assign codes. Two or more investigators will independently code each interview/focus group. Additional codes will be iteratively added in an inductive fashion following coder consensus. We will analyze PL paradata metrics using Google Analytics to characterize user activity for multiple features over the study period (eg, community message board, daily check-ins, provider messaging), as well as establish any associations between frequency/dose of user activity and differences in primary outcomes.

Descriptive statistics will be used to analyze the proportions of patients and providers completing implementation steps (eg, reach, adoption dimensions of RE-AIM). Pearson correlation and binary logistic regression will be used to for exploratory analysis of associations between patient-specific covariates (eg, demographics) and outcome measures for each dimension (eg, reach, including PL usage by patients). We will also examine associations between site-specific covariates (reach measures attained for their patients, adoption measures achieved for their providers, site-level measures, for example, organizational readiness), and differences in patients’ primary outcomes (viral suppression, retention in care) observed on average for sites randomized to PL*.* Provider survey responses will be analyzed using descriptive statistics for Likert responses and with qualitative analysis, as described before for open-ended responses.

## Results

### Formative Phase

Interviews and focus groups for the formative phase were completed between April and December 2021. Qualitative analysis of interview and focus group transcripts is ongoing as of March 2022. Preliminary app modification requests have been finalized following consensus reached between study team members and the DC Cohort Executive Committee, and the first round of user testing of the modified app with people with HIV has been completed. Recruitment for beta testing with people with HIV was initiated in February 2022.

### Cluster Randomized Controlled Trial

Randomization will occur in spring 2022. Patient recruitment for the cluster randomized controlled trial is planned to start in spring 2022.

### Implementation Evaluation

Following completion of the preimplementation evaluation, a series of instruments were designed using the RE-AIM framework to support planned data collection for relevant implementation outcomes during the mid- and postimplementation evaluations. The preimplementation evaluation process, including the design of instruments guided by several relevant implementation science frameworks, and logistical planning surrounding the method and timing of instrument distribution, data export/access, and analysis in conjunction with trial activities, will be described in further detail in a separate publication.

We have previously applied the CFIR toward examination of PL implementation in another cohort seeking care at a Ryan White Clinic in Virginia, using a rigorous process of in-depth interviews with participating stakeholders [55]. CFIR domains (with corresponding constructs) that emerged from our prior evaluation as most relevant to PL implementation included Inner Setting (Compatibility, Access to Knowledge and Information), Outer Setting (Patient Needs and Resources, External Policy and Incentives), Characteristics of Individuals (Knowledge and Beliefs), Innovation Characteristics (Adaptability, Complexity), and Implementation Process (Planning, Engagement of Key Stakeholders). Provider surveys (provider baseline and follow-up surveys) were designed to incorporate items that assess these constructs.

## Discussion

### Principal Results

This is a novel, large-scale cluster randomized controlled trial with a hybrid efficacy-implementation design examining the impact of the PL mHealth intervention on HIV-related patient outcomes with a direct comparison arm of usual care. Prior work to evaluate the clinical effectiveness of PL has been limited to single-arm prospective cohort studies within a nonurban population in Central Virginia. This project will use a randomized design to test the effectiveness of PL against usual care in a diverse urban cohort of people with HIV not achieving durable viral suppression or retention in care. We hypothesize that compared to usual care, clusters participating in the PL intervention will experience improved rates in viral suppression, visit constancy, and retention in care at 12 months.

### Comparison With Prior Work

This study will significantly extend the evidence base for this intervention beyond more rural samples by testing its efficacy in a vulnerable urban sample using a robust study design. Further, evaluation of PL implementation to date has been limited to a small subset of clinics within a nonurban context. This project builds on prior preliminary work using implementation science frameworks to identify best practices for implementing PL in a range of different urban HIV care settings and corresponding determinants of implementation success and will inform future disseminations of PL and other mHealth tools at scale in order to improve the lives and health of people with HIV.

### Limitations

Limitations exist in this study design. Although formative work for this distinct patient population to date does not suggest significant changes to the platform will be required based on preliminary review, beta testing is underway and further modifications may be suggested by users. The study research and development teams will prioritize the most feasible changes prior to trial initiation. DC Cohort sites are heterogenous in the services provided to support patient adherence and retention and the patient populations they primarily serve, presenting a potential challenge in assessment of the impact of PL when implemented in conjunction with variable services across clinics. The usual care condition will require assessment at baseline and periodically throughout the trial, and site-level characteristics must be adjusted for during statistical analyses.

### Conclusion

Output from the formative phase is currently being analyzed, and corresponding preliminary modifications to the platform are being tested by people with HIV within the DC Cohort. Modifications will be finalized by the app development team following beta testing. Trial activities are expected to begin in spring 2022.

## Appendix

Multimedia Appendix 1 CONSORT-EHEALTH checklist (V 1.6.1).

## Abbreviations

ART: antiretroviral therapy
CD: cluster of differentiation
CFAR: Center for AIDS Research
CFIR: Consolidated Framework for Intervention Research
DC: Washington, DC
DOH: Department of Health
HRSA: Health Resources and Services Administration
MEM: mixed effects model
mHealth: mobile health
NIH: National Institutes of Health
ORIC: Organizational Readiness for Implementing Change
PI: principal investigator
PL: PositiveLinks
RA: research assistant
RE-AIM: Reach Effectiveness Adoption Implementation Maintenance
